# Role of Bruton’s tyrosine kinase in B cells and malignancies

**DOI:** 10.1186/s12943-018-0779-z

**Published:** 2018-02-19

**Authors:** Simar Pal Singh, Floris Dammeijer, Rudi W. Hendriks

**Affiliations:** 1 Department of Pulmonary Medicine, Room Ee2251a, Erasmus MC Rotterdam, PO Box 2040, NL 3000 CA Rotterdam, The Netherlands; 2 Department of Immunology, Rotterdam, The Netherlands; 3 Post graduate school Molecular Medicine, Rotterdam, The Netherlands; 4 Erasmus MC Cancer Institute, Erasmus MC, Rotterdam, The Netherlands

**Keywords:** B cell development, B cell receptor signaling, Bruton’s tyrosine kinase, Chemokine receptor, Chronic lymphocytic leukemia, Ibrutinib, Leukemia, Lymphoma, Tumor microenvironment

## Abstract

Bruton’s tyrosine kinase (BTK) is a non-receptor kinase that plays a crucial role in oncogenic signaling that is critical for proliferation and survival of leukemic cells in many B cell malignancies. BTK was initially shown to be defective in the primary immunodeficiency X-linked agammaglobulinemia (XLA) and is essential both for B cell development and function of mature B cells. Shortly after its discovery, BTK was placed in the signal transduction pathway downstream of the B cell antigen receptor (BCR). More recently, small-molecule inhibitors of this kinase have shown excellent anti-tumor activity, first in animal models and subsequently in clinical studies. In particular, the orally administered irreversible BTK inhibitor ibrutinib is associated with high response rates in patients with relapsed/refractory chronic lymphocytic leukemia (CLL) and mantle-cell lymphoma (MCL), including patients with high-risk genetic lesions. Because ibrutinib is generally well tolerated and shows durable single-agent efficacy, it was rapidly approved for first-line treatment of patients with CLL in 2016. To date, evidence is accumulating for efficacy of ibrutinib in various other B cell malignancies. BTK inhibition has molecular effects beyond its classic role in BCR signaling. These involve B cell-intrinsic signaling pathways central to cellular survival, proliferation or retention in supportive lymphoid niches. Moreover, BTK functions in several myeloid cell populations representing important components of the tumor microenvironment. As a result, there is currently a considerable interest in BTK inhibition as an anti-cancer therapy, not only in B cell malignancies but also in solid tumors. Efficacy of BTK inhibition as a single agent therapy is strong, but resistance may develop, fueling the development of combination therapies that improve clinical responses. In this review, we discuss the role of BTK in B cell differentiation and B cell malignancies and highlight the importance of BTK inhibition in cancer therapy.

## Background

Protein kinases represent classes of enzymes that catalyze phosphorylation of proteins and thereby alter their substrate’s activity or capacity to interact with other proteins. Kinase signaling pathways represent the most common form of reversible post-translational modifications that control many aspects of cellular function. Aberrant activation of protein kinases drive major hallmarks of malignancies, including alterations in cellular proliferation, survival, motility and metabolism, as well as angiogenesis and evasion of the anti-tumor immune response [1, 2].

One such kinase that plays a crucial role in oncogenic signaling is Bruton’s tyrosine kinase (BTK), which is critical for the survival of leukemic cells in various B cell malignancies. BTK was initially shown to be mutated in the primary immunodeficiency X-linked agammaglobulinemia (XLA) and is essential at various stages of B lymphocyte development [3, 4]. XLA is an inherited immunodeficiency disease originally described by the pediatrician Ogdon Bruton in 1952 and characterized by recurrent bacterial infections. Due to a severe block of B cell development in the bone marrow, XLA patients have very low numbers of B cells in the circulation and antibodies are almost completely absent in the serum. A milder phenotype of the disease is present in CBA/N mice, which harbor the loss-of-function mutation R28C BTK [5, 6]. These mice, known as *xid* (X-linked immunodeficiency) mice, manifest only minor defects in B cell development in the bone marrow, but instead the differentiation and survival of mature peripheral B cells is severely impaired [7–10]. Importantly, BTK has received large interest since small-molecule inhibitors of this kinase have shown excellent anti-tumor activity in clinical studies [11, 12]. In particular, the orally administered BTK inhibitor ibrutinib, which forms a covalent bond with a cysteine residue in the BTK active site, was also approved for first-line treatment of patients with chronic lymphocytic leukemia (CLL) and small lymphocytic leukemia (SLL) in 2016 [13].

Shortly after its discovery as the non-receptor tyrosine kinase defective in XLA [3, 4], BTK was placed in the signal transduction pathway downstream of the B cell receptor (BCR). This receptor is expressed on the B cell surface and has the unique capacity to specifically recognize antigens due to hypervariable regions present in the immunoglobulin heavy (IGH) and light (IGL) chains that together form the BCR [14]. BTK is also involved in many other signaling pathways in B cells, including chemokine receptor, Toll-like receptor (TLR) and Fc receptor signaling. Expression of BTK is not restricted to B cells, as also cells of the myeloid lineage express BTK. In these cells, BTK acts also downstream of TLRs and e.g. the FcεR in mast cells [15, 16] and the FcyRI in macrophages [17, 18]. In addition, BTK is involved in various other pathways, including Receptor activator of nuclear factor-κB (RANK) in osteoclasts [19], collagen and CD32 signaling in platelets [20] and the NLRP3 inflammasome in macrophages and neutrophils [21]. Since myeloid cells are important components of the tumor microenvironment and particularly tumor-associated macrophages contribute to cancer progression [22, 23], there is currently a considerable interest in BTK inhibition as an anti-cancer therapy not only in B cell leukemias but also in other hematological malignancies and solid tumors [24–27].

In this review, we describe the importance of BTK in multiple signaling pathways. We discuss the crucial function of BTK in different stages of normal B cell development. In addition, we discuss its role in oncogenic signaling in B cell malignancies associated with genetic events that result in increased BTK activity. We describe clinical benefits of targeting BTK with small molecule inhibitors in B cell malignancies. Finally, we discuss the effects of BTK inhibitors on tumor growth in solid malignancies in the context of the function of myeloid cells in the tumor environment.

## BTK structure

**Fig. 1:**
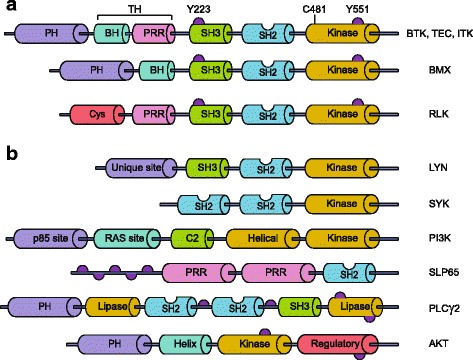
Domain structure of TEC kinase family members and key interacting partners of Bruton’s tyrosine kinase. **a** Schematic overview of the protein structure of BTK and other TEC kinase family members. Shown are five different domains, as explained in text, the Y223 autophosphorylation site, the Y551 phosphorylation site that activates BTK, and the C481 binding site of ibrutinib. **b** Schematic overview of the protein structure of key interacting partners of BTK. PH, pleckstrin homology; TH, TEC homology; BH, BTK homology; PRR, proline rich domain; SH2/SH3, SRC homology domains 2 and 3; Cys, cysteine-string motif

BTK is one of the five members of the TEC family of non-receptor tyrosine kinases - along with tyrosine kinase expressed in hepatocellular carcinoma (TEC), interleukin-2-inducible T cell kinase (ITK), resting lymphocyte kinase (RLK) and bone marrow expressed kinase (BMX) - which are strongly conserved throughout evolution [28]. BTK, TEC and ITK are most similar and both contain five different protein interaction domains (Fig. 1a). These domains include an amino terminal pleckstrin homology (PH) domain, a proline-rich TEC homology (TH) domain, SRC homology (SH) domains SH2 and SH3, as well as kinase domain with enzymatic activity [28, 29]. BTK is essentially cytoplasmic and is only transiently recruited to the membrane through interaction of its PH domain with phosphatidylinositol-3,4,5-triphosphate (PIP_3_), which is generated by phosphatidylinositol-3 kinase (PI3K) (Fig. 1b) [14]. BTK activation occurs in two steps upon its recruitment to the cell membrane. First, BTK is phosphorylated at position Y551 in the kinase domain by SYK or SRC family kinases [30]. Phosphorylation of BTK at Y551 promotes its catalytic activity and subsequently results in its autophosphorylation at position Y223 in the SH3 domain [31]. Phosphorylation at Y223 is thought to stabilize the active conformation and fully activate BTK kinase activity [32]. Nevertheless, a Y223F mutation did not significantly affect the function of BTK during B cell development in vivo, since B-cell specific transgenic expression of Y223F-BTK could still rescue the *xid* phenotype of Btk-deficient mice [33]. Therefore, the function of the Y223 BTK autophosphorylation site remains unclear in B cells and to date is unexplored in vivo in myeloid cells.

## BTK in B cell receptor signaling

The IgM BCR is essential for survival of peripheral B cells [34]. In the absence of BTK B cells have a high rate of apoptosis, which correlates with strongly reduced BCR-mediated induction of the anti-apoptotic protein Bcl-xL [35, 36]. Upon stimulation with anti-IgM, cell size enlargement and degradation of the cyclin inhibitor p27Kip1 occurs normally, indicating that BTK is not essential for several G1 events [37]. BTK-deficient B cells enter early G1, but not S phase of the cell cycle, because they fail to induce cyclin D2 expression [38]. Apart from B cell survival and proliferation, the BCR controls integrin α4β1 (VLA-4)-mediated adhesion of B cells to vascular cell adhesion molecule-1 (VCAM-1) and fibronectin via BTK [39].

BCR cross-linking activates four families of non-receptor protein tyrosine kinases and these are transducers of signaling events including phospholipase Cγ (PLCγ), mitogen-activated protein kinase (MAPK) activation, nuclear factor kappa-light-chain-enhancer of activated B cells (NF-кB) pathway components and activation of the serine/threonine kinase AKT (or protein kinase B, PKB).

**Fig. 2:**
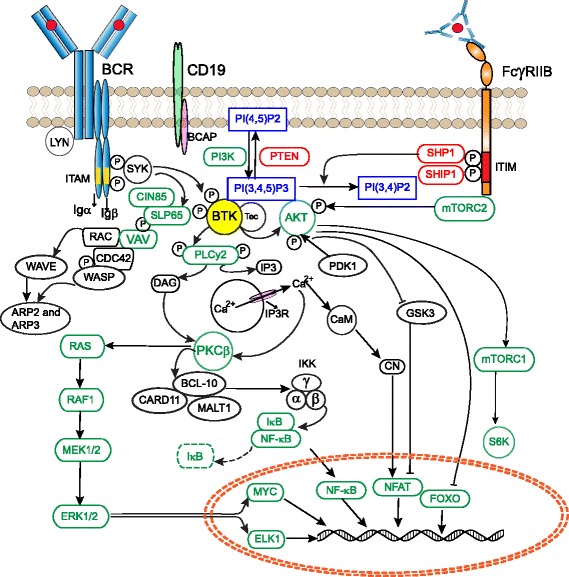
Role of Bruton’s tyrosine kinase downstream of the B cell receptor. Signaling cascade showing important events downstream of B cell receptor (BCR). Antigen engagement by the BCR results in the formation of a micro-signalosome whereby BTK activates four families of non-receptor protein tyrosine kinases that transduce key signaling events including phospholipase Cγ, mitogen-activated protein kinase (MAPK) activation, nuclear factor kappa-light-chain-enhancer of activated B cells (NF-кB) pathway components and activation of the serine/threonine kinase AKT (PKB). In addition, BTK mediated signaling events are regulated by various phosphatases that can be recruited to the cell membrane, following crosslinking of inhibitory receptors, e.g., FcγRIIB that is exclusively expressed on B cells and signals upon immune complex binding. See text for details

The IgM BCR has a very short cytoplasmic domain and thus cannot signal directly, but associates with the disulphide-linked Ig-α/Ig-β(CD79a/CD79b) heterodimers. These transmembrane proteins contain immunoreceptor tyrosine based activation motifs (ITAMs) in their cytoplasmic domain (Fig. 2). BCR engagement by antigen induces ITAM phosphorylation by Src-family protein tyrosine kinases such as LYN, thereby creating docking sites for spleen tyrosine kinase (SYK)(Fig. 1b) [40]. In addition, LYN and SYK also phosphorylate tyrosine residues in the cytoplasmic tail of the B-cell co-receptor CD19 and/or the adaptor protein B-cell PI3K adaptor (BCAP), which facilitates recruitment and activation of PI3K and the guanine nucleotide exchange factor VAV [41, 42]. VAV further enhances enzymatic activity of PI3K through activation of RAC1, a member of Rho family of GTPases [43]. PI3K phosphorylates PIP2 to generate PIP3, which acts as a critical secondary messenger for activating downstream pathways. PIP3 interacts with the BTK PH-domain, resulting in its recruitment to the plasma membrane [44].

In addition, Ig-α contains a conserved non-ITAM tyrosine residue, Y204, that upon activation by SYK recruits and phosphorylates the central B cell-linker molecule SH2-domain-containing leukocyte protein of 65 kDa (SLP65/BLNK) [45] (Fig. 2). Hereby, the adaptor molecule Cbl-interacting protein of 85 kD (CIN85) functions to oligomerize SLP65 and assembles intracellular signaling clusters for B cell activation [46]. SLP65 serves as a scaffold for various signaling molecules, including BTK and its substrate PLCγ2 [47–50]. In this micro-signalosome BTK is activated through Y551 phosphorylation by SYK or LYN and subsequently at Y223, as described above [30–32]. Fully activated BTK phosphorylates PLCγ2 at Y753 and Y759, which is important for its lipase activity [51]. Activated PLCγ2 hydrolyses PIP2 into inositol triphosphate (IP3) and diacylglycerol (DAG). IP3 regulates intracellular calcium levels and thereby activates nuclear factor of activated T cells (NFAT) transcription, via calcineurin and calmodulin. DAG mediates activation of protein kinase Cβ (PKCβ), which induces activation of several members of the MAPK family, including extracellular signal-regulated kinases 1 and 2 (ERK1/ERK2) and other MAPK targets, such as Jun N-terminal kinase (JNK), p38, and NF-кB pathway components [52] (Fig. 2). Hereby, BTK links the BCR to NF-кB activation [53, 54].

Another important branching point is induced more upstream in the BCR signaling cascade: in addition to BTK, PIP3 also interacts with PH-domain of AKT, resulting in its recruitment to the plasma membrane. Full activation of AKT requires phosphorylation at position T308, induced by 3-phosphoinositide-dependent protein kinase-1 (PDK1), and at S473, phosphorylated by mechanistic target of rapamycin (mTOR) complex 2 (See Ref [55] for an excellent review). Fully activated AKT then returns to the cytoplasm to enable a pro-survival signaling program that involves NFAT, forkhead transcription factors (FOXOs) and NF-кB-mediated pathways. Importantly, phosphorylation of AKT is positively regulated by BTK [56]. The BTK family member TEC, which can partly compensate for BTK [57], may on the other hand limit the capacity of BTK to activate AKT [58].

Upon activation in germinal centers (GCs), B cells can perform IGH chain class switching, by which it changes Ig expression from one isotype to another with different effector function, e.g. from IgM to IgG. In this process, the IGH constant (C) region is changed, but the variable (V) region remains the same. Interestingly, in contrast to IgM, the IgG BCR contains a cytoplasmic domain of considerable length with an Ig tail tyrosine (ITT) motif, which amplifies signaling [59]. SYK is required for ITT phosphorylation followed by recruitment of BTK through the adapter protein Grb2, leading to enhancement of IgG BCR-induced calcium mobilization. This amplification loop is thought to represent a cell-intrinsic mechanism for rapid activation of class-switched memory B cells.

## Regulation of BTK activity and expression

Consistent with its crucial role in B cell differentiation, proliferation and survival, proper control of BTK activity is important for B cell homeostasis. Several mechanisms for its regulation have been identified to date.

The recruitment of BTK to the plasma membrane and its subsequent activation is regulated by various phosphatases that can be recruited to the cell membrane, similar to BTK. For example, the FcγRIIB is an inhibitory receptor that is exclusively expressed on B cells [60]. In contrast to the Igα/Ig-β ITAM motifs, FcγRIIB has immune tyrosine inhibitory motifs (ITIMs) in its cytoplasmic domain [61, 62] (Fig. 2). The binding of IgG antibodies to FcγRIIB results in LYN-mediated phosphorylation of ITIMs and recruitment of protein phosphatases such as SH2-domain containing inositol polyphosphate 5’phosphatase-1 (SHIP1) [63–65]. SHIP1 catalyzes the dephosphorylation of PIP3 and thereby inhibits recruitment of PH-domain containing proteins, such as BTK and PLCγ2 to the cell membrane. As a result, the downstream increase in intracellular calcium levels is diminished. Another phosphatase, SH2 domain containing protein tyrosine phosphatase-1 (SHP1), has the capacity to dephosphorylate tyrosine on BTK [65]. SHP1 acts downstream of CD22, a lectin molecule, and the glycoprotein CD5, both of which are on the B cell surface and function as negative regulators of BCR signaling.

In addition, several negative regulators of BTK have been identified. The iBTK protein directly binds to the BTK PH domain and thereby inhibits its activity [66]; PKCβ phosphorylates BTK on residue S180 in TH domain, modulating its membrane localization [67]; microRNA-185 reduces BTK mRNA levels and thereby downregulates BTK expression [68]. Likewise, expression of other microRNAs, including miR-210 and miR-425, significantly reduce BTK expression [69]. In this context, it was shown that treatment of primary CLL samples with histone deacetylase (HDAC) inhibitors resulted in increased expression of these miRs and decreased BTK protein. On the other hand, BTK itself can initiate a proteasome-dependent positive autoregulatory feedback loop by stimulating transcription from its own promoter through a pathway involving NF-кB [70].

## BTK in other signaling pathways

### Chemokine receptors

**Fig. 3:**
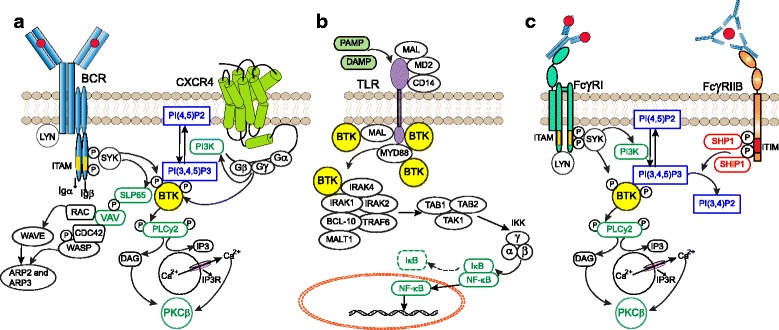
Role of Bruton’s tyrosine kinase downstream of chemokine receptors, Toll-like receptors and activating Fcγ receptors. Signaling cascade showing important events downstream of (**a**) Chemokine receptors (e.g. CXCR4): upon chemokine binding to the extracellular domain Gα and Gβy subunits can independently activate PI3K, which results in activation of BTK, AKT and MAPK-dependent pathways. **b** Toll-like receptors: upon ligand recognition TLRs recruit different proteins including TIR, MYD88, IRAK1 and TIRAP/MAL, all of which interact with BTK and induce downstream activation of the transcription factor NF-κB. **c** Activating Fc receptors (e.g. FcγRI): Following FcγRI cross-linking, Src-kinases, SYK, PI3K-γ and BTK are activated. In contrast, inhibitory Fc-receptors (FcγRIIB) containing ITIM domains recruit phosphatases and reduce BTK activation (Fig. 2). See text for details

These receptors are G-protein coupled receptors that consist of seven transmembrane spanning domains and intracellular hetero-trimeric G-proteins composed of α, β, and y subunits (Gα, Gβ, and Gy) [71]. The chemokine receptors CXCR4 and CXCR5 are expressed on B cells in different stages of their development and play important roles in trafficking, homing and homeostasis [72]. Chemokine binding to the extracellular domain of its receptor induces conformational changes that result in dissociation of Gα and Gβy subunits (Fig. 3a). Both Gα and Gβy subunits can independently activate PI3K, which results in activation of BTK, AKT and MAPK dependent pathways [73, 74]. In addition, both Gα and Gβy subunits can directly bind BTK via the PH and TH domain [74, 75]. It has been shown that the Gα subunit directly stimulates the activity of BTK [76]. Due to its function downstream of chemokine receptors including CXCR4 and CXCR5, BTK is important for positioning of B cells in various lymphoid tissue compartments. This was first demonstrated by adoptive transfer experiments with BTK-deficient B cells, which exhibited impaired in vivo migration and homing to lymph nodes [77].

#### Toll-like receptors (TLRs)

These extracellular or intracellular pattern recognition receptors are characterized by leucine-rich repeats and Toll/interleukin-1 receptor (TIR) domains (Fig. 3b). TLRs, expressed in B cells or myeloid cells, recognize structurally conserved molecules derived from bacteria and viruses. Upon activation most TLRs recruit the adaptor myeloid differentiation primary response 88 (MYD88) [78]. MYD88 activates interleukin-1 receptor-associated kinase1 (IRAK1), either on its own or in combination with an adaptor molecule, TIR domain containing adaptor protein (TIRAP, also known as MyD88 adapter-like (MAL)). BTK interacts with four different proteins downstream of TLR signaling including TIR, MYD88, IRAK1 and TIRAP/MAL) [79–81]. TLR signaling induces transcription factors including NF-кB, activator protein-1 (AP-1) and interferon regulatory factor 3 (IRF3), which results in activation, proliferation, antibody secretion, class switch recombination and pro-inflammatory cytokine production in B cells.

#### Fc receptor signaling

BTK is involved in signaling of both activating (ITAM-containing) and inhibitory (ITIM-containing) Fc-receptors, whose balance regulates several myeloid cell processes including activation, polarization and phagocytosis (Fig. 3c) [60, 82]. BTK is rapidly activated upon FcεRI cross-linking in mast cells [15]. In parallel to BCR signaling, following activating Fc-receptor cross-linking, SRC-kinases, SYK, PI3K-γ and BTK are activated [60]. In contrast, inhibitory Fc-receptors (FcγRIIB) containing ITIM domains recruit phosphatases and reduce BTK activation (see above).

## BTK and B cell development in the bone marrow

**Fig. 4:**
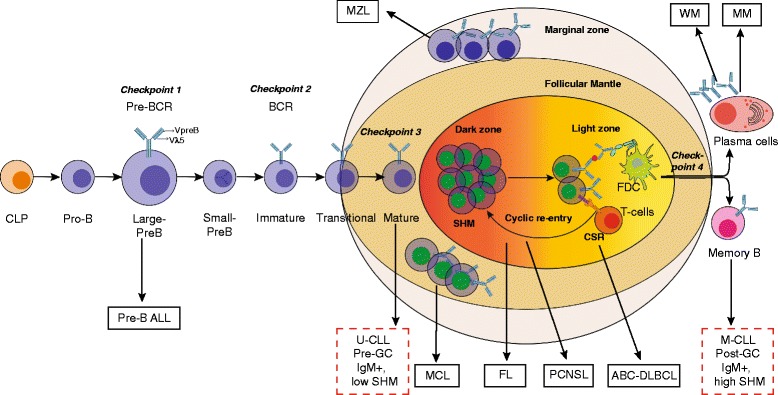
Stages of B cell differentiation and associated malignancies. Model of B cell development indicating different stages of B cell differentiation and important immune checkpoints where BTK plays a key role. Various B-cell malignancies are indicated, which are associated with abnormal BTK signaling at distinct stages of B-cell differentiation and activation. Note that the cellular origin of U-CLL is thought to be CD5^+^ mature B cells. Somatic hypermutation status of BCR and gene expression profiling indicates post-germinal center (GC) origin of M-CLL. See text for detailed information. CLP, common lymphoid progenitor; CSR, class switch recombination; FDC, follicular dendritic cell; SHM, somatic hypermutation

Even before the gene involved in XLA was identified, X-chromosome inactivation studies showed that the defect in XLA patients was intrinsic to the B cell lineage and that myeloid cells had no developmental defects [83, 84]. B cells are generated from hematopoietic stem cells in the bone marrow throughout life by the ordered rearrangement of IGH and IGL chain gene segments (Fig. 4). After productive recombination of the IGH V, D and J genes, the IGH μ protein is expressed on the cell surface in association with the two invariant surrogate light chain (SLC) proteins VpreB and λ5 [85, 86],as the pre-BCR. Pre-BCR signaling marks a crucial checkpoint (*checkpoint 1*) to test the functionality of the IGH μ protein (Fig. 4) [87, 88]. To date, the mechanisms that initiate pre-BCR-mediated signaling are not fully resolved as both cell-autonomous and ligand-mediated signaling has been described [89–92]. An important function of pre-BCR signaling is to inhibit further IGH VDJ recombination, a phenomenon known as allelic exclusion [88]. Pre-BCR signaling leads to proliferation of pre-B cells and at the same time downregulation of SLC expression [88]. This is important for the exit of pre-B cells from the cell cycle to undergo the transition from large, cycling cells into small resting pre-B cells, in which IGL chain recombination occurs. In XLA patients B cell development is almost completely arrested at the pre-B cell stage. Although pre-B cells expressing intracellular IGH μ are present, they are small in size, indicating that BTK is essential for pre-BCR-dependent proliferation. BTK-deficient mice have only a mild pre-B cell defect, whereby pre-B cells show impaired developmental progression into immature B-cells [9, 10]. Nevertheless, an almost complete block is only found in mice that are double-deficient for e.g. BTK and SLP65 or BTK and TEC [57, 93, 94]. Interestingly SLP65-deficient mice, which also have a mild arrest at the pre-B cell stage, develop pre-B cell leukemia resembling pre-B ALL in humans [93, 94]. In this regard, BTK cooperates with SLP65 as a tumor suppressor independent of its kinase activity [95, 96]. SLP65 also mediates downregulation of SLC expression [97]. Analyses in wild-type, BTK and SLP65 deficient pre-B cells demonstrated that pre-BCR signaling induces *IGL* κ locus accessibility by functional redistribution of enhancer-mediated chromatin interactions [98]. BTK and SLP65 are important for the induction of *IGL* chain germ-line transcripts that are associated with locus accessibility. Moreover, BTK-deficient mice exhibit a ~ 50% reduction of *IGL* κ chain usage [98, 99]. Transcriptome analyses showed that BTK/SLP65deficient pre-B cells fail to efficiently upregulate many genes involved in *IGL* chain recombination, including *Aiolos, Ikaros, Spib, Irf4, Oct2, polymerase-μ, and Mbp-1* [98].

If *IGL* chain recombination is not productive or the resulting BCR is autoreactive (*checkpoint 2*) (Fig. 4), developing B cells will undergo secondary *IGL* chain rearrangements, a process termed receptor editing [100–102]. Many autoreactive B cells are lost during development to the immature IgM^+^ B cell stage (central B cell tolerance), but it has been estimated that ~ 40% of the newly formed B cells that leave the bone marrow have self-reactivity [92].

## BTK and peripheral B cell development and activation

Immature B cells from the bone marrow migrate to the spleen, where selection and maturation is continued within the transitional B cell compartment containing T1 and T2 B cells. In mice, T1 B cells, but not T2 B cells, are very sensitive to BCR-mediated apoptosis, indicating that the T1 to T2 differentiation marks a peripheral tolerance checkpoint (*checkpoint 3*) [103, 104]. In the absence of BTK, T2 cells do not generate survival responses and peripheral B cells are reduced by ~ 50%. As a result, BTK-deficient B cells exhibit an impaired transition from IgM^high^IgD^low^ into IgM^low^IgD^high^ mature B cells. BTK-deficient mice lack the population of innate-like CD5^+^ B-1 cells, present in the peritoneal and pleural cavities and in small proportions in the spleen [7–9]. Consistent with the finding that these cells are important for IgM and IgG3 levels in the serum, in BTK-deficient mice IgM and IgG3 levels in serum are severely reduced, but the other isotypes are largely normal.

Marginal zone B cells are present in an area at the outermost portion of the white pulp in the spleen and are phenotypically defined as IgM^hi^IgD^lo^CD21^high^CD23^low^ B cells that respond to polysaccharide antigens independently of T cell help (Fig. 4). BCR and NOTCH2 signaling determine whether T1 B cells expressing surface ADAM10 are committed to becoming MZ B cells in vivo in the spleen [105, 106]. Although contradictory findings on the numbers of MZ B cells in BTK-deficient mice have been reported, it is clear that developing BTK-deficient MZ B cells have a selective disadvantage [107, 108].

Upon antigen recognition, activated B cells may either go into an extrafollicular response or develop into GC B cells [109, 110]. In the GCs B cells strongly proliferate and undergo somatic hypermutation (SHM) induced by activation induced cytidine deaminase (AID). GC B cells are selected involving follicular dendritic cells (FDCs) and T-follicular helper (T_FH_) cells (*checkpoint 4*) based on their antigen affinity [109]. Although BTK-deficient mice show normal T-cell dependent responses to model antigens, such as TNP-KLH [7, 8], there is a significant reduction in GC B cell numbers in physiological models, e.g. influenza virus infection [108]. In this context, it is of note that mice expressing the constitutively active BTK mutant E41K fail to form GCs [111, 112], whereas overexpression of wild-type BTK induces spontaneous GC formation [113, 114]. Consequently, BTK-overexpressing mice develop autoimmunity involving B cell-induced disruption of T cell homeostasis [113, 114].

## BTK in B cell malignancies

BTK activity is crucial for survival and proliferation of leukemic B cells and for their interactions with cells in the tumor microenvironment. Below, we discuss the role of BTK in various B cell malignancies (Fig. 4).

### CLL

This is the most common leukemia in the western world, primarily affecting the elderly, and is characterized by the accumulation of mature circulating IgM^low^ CD5^+^ B cells [115]. Several genetic aberrations with prognostic value and impact on treatment decisions in CLL have been described. These include deletions of the chromosomal regions 17p13 (containing the *TP53* tumor suppressor gene), 11q23 (containing DNA damage checkpoint protein *ATM*), or 13q14 (miR-15a, miR-16-1), and trisomy of chromosome 12 [116, 117]. Furthermore, > 80% of cases harboring del(17p) also carry *TP53* mutations in the remaining allele [118]. Such patients with *TP53* defects are classified as ‘high-risk’ and often respond poorly to therapy [119]. Moreover, a significant proportion of CLL patients carry a TP53 mutation in the absence of a 17p deletion [120, 121].

On the basis of SHM status of IGHV, CLL can be grouped into mutated CLL (M-CLL) and unmutated CLL (U-CLL). M-CLL have a more favorable prognosis and are derived from post-GC B cells. The origin of U-CLL appeared less clear and several cellular origins of CLL were suggested, including MZ B cells, CD5^+^ B cells, and regulatory B cells [122–126]. Although initial gene expression profiling indicated that M-CLL and U-CLL were quite homogeneous and related to memory B cells derived from T cell-dependent and T-cell independent responses, respectively [123], more recent gene expression profiling studies have provided evidence for a different origin [124]. This study by Seifert et al. shows that U-CLL derives from unmutated mature CD5^+^ B cells. Moreover, it was concluded that M-CLL originate from a distinct and previously unrecognized post-GC B cell subset with a CD5 + CD27+ surface phenotype.

Several lines of evidence establish a role of chronic BCR-mediated signaling in CLL pathogenesis [127]. (i) Prognosis is correlated with the BCR SHM status [128]; (ii) The BCR repertoire is highly restricted [129, 130], suggesting a role for antigenic selection in the initiation or progression of CLL. Antigens binding to CLL BCRs include self-antigens, such as non-muscle myosin IIA, vimentin, apoptotic cells and oxidized low-density lipoprotein [131–136], as well as foreign antigens (bacterial polysaccharides and β-(1,6)-glucan, a major antigenic determinant on fungi [132–137]); Interestingly, evidence was provided in mice that pathogens may drive CLL pathogenesis by selecting and expanding pathogen-specific B cells that cross-react with self-antigens [138]; (iii) CLL cells were reported to display cell-autonomous Ca^2+^ mobilization in the absence of exogenous ligands, by virtue of recognizing a single conserved BCR-internal epitope in the IGHV second framework region [139]; very recently, it was found that the internal epitopes recognized by CLL BCRs from distinct subgroups are different [140]. Moreover, the avidity of the BCR-BCR interactions that can lead to receptor declustering influences the clinical course of the disease [139, 140].

In line with chronic BCR-mediated signaling, CLL cells show constitutive activation of various BCR pathway associated kinases. Hereby, BTK is essential for constitutively active pathways implicated in CLL cell survival, including AKT, ERK and NF-кB, both in patient cells and mouse models [133, 141–143]. CLL cells are thought to interact with the tissue microenvironment and lymph node resident CLL cells show gene expression signatures indicative of BCR activation [144, 145]. Moreover, BTK is critical for BCR- and chemokine-controlled integrin-mediated retention and/or homing of CLL B cells in their microenvironment [146].

### Mantle cell lymphoma (MCL)

This disease results from malignant transformation of B lymphocytes in the mantle zones surrounding GCs (Fig. 4) and has a remarkably biased BCR repertoire [147]. Approximately 85% of the patients harbor the hallmark chromosomal translocation t(11:14)(q13;32). This event juxtaposes the *CCND1* gene to an enhancer in the Ig heavy chain locus [148], resulting in constitutively cyclin-D1 expression and abnormal proliferation. In a fraction of MCL patients lymphoma cells express the SOX11 transcription factor, which is associated with minimal Ig SHM, higher genetic instability and a more aggressive clinical course [149, 150]. Primary MCL cells show strong expression and Y223-phosphoryation of BTK [151] and in a subset of patients constitutive phosphorylation of LYN, SLP65, SYK and PKCβ [152, 153]. Similar to CLL, the tumor microenvironment plays an important role in MCL pathogenesis. BTK is essential for retention of MCL cells in lymphoid tissues, since BTK inhibition induces an egress of malignant cells into peripheral blood [154].

### Waldenström’s Macroglobulinemia (WM)

This indolent B-cell malignancy is characterized by IgM-secreting lymphoma cells in the bone marrow. The majority of WM patients have a somatic leucine to proline substitution at position 265 of MyD88 (MyD88^L265P^) [155]. This activating mutation has also been reported in low frequencies in activated B-cell-like diffuse large B-cell lymphoma (14%–29%) (see below), primary central nervous system lymphoma (PCNSL; 33%), mucosa-associated lymphoid tissue (MALT) lymphoma (9%), and CLL (2.9%) [156–159]. The mutated MyD88^L265P^ protein binds phosphorylated-BTK and triggers NF-кB signaling [160]. In addition, ~ 30% of WM patients show the CXCR4 S338X somatic mutation, leading to enhanced CXCL12-triggered activation of AKT and ERK [161]. In this regard, CXCR4 and VLA-4 interactions have been shown to regulate trafficking and adhesion of WM cells to the bone marrow [162].

### ABC-DLBCL

DLBCL is the most common form of B cell non-Hodgkin lymphomas (B-NHLs) representing ~ 30–40% of all cases. Patients most often present with a rapidly growing tumor in single or multiple, nodal or extranodal sites. Based on gene expression profiling, three major molecular subtypes have been identified: GC B-cell-like (GCB-DLBLCL), activated-B-cell-like (ABC-DLBCL) and primary mediastinal B-cell lymphoma (PMBL) [163]. Whereas GCB-DLBCL and ABC-DLBCL make up the majority of cases at roughly equal frequency, PMBL accounts for up to 10% of cases of DLBCL [164]. GCB-DLBCL tumors express many genes found in normal GC B cells and have typically switched to an IgG BCR, while gene expression in ABC-DLBCL, which are predominantly IgM^+^, resembles that of antigen-activated plasmablasts [165, 166]. ABC-DLBCL has an inferior clinical outcome than GCB-DLBCL with a three-year overall survival of ~ 45% [167].

ABC-DLBCL are dependent on constitutive NF-кB signaling for their survival and proliferation [168–170]. Approximately 50% of ABC-DLBCL harbor mutations in CARD11 or other NF-кB pathway components, including the MyD88^L265P^ mutation [169–171]. In addition, ~ 20% of patients carry an activating mutation in CD79A/B. Consistent with a role of NF-кB downstream of the BCR (Fig. 2), it was found that knockdown of BCR components, CD79A/B and downstream signaling molecules, induced cell death in ABC-DLBCL lines with unmutated CARD11 [172]. Moreover, RNAi experiments demonstrated that ABC-DLBCL lines are dependent on MyD88 and its associated kinase IRAK1 for their survival in line with NF-kB function in the TLR pathway (Fig. 3b). In addition, SYK amplification and deletion of PTEN, a phosphatase that dephosphorylated PIP_3_, are also selective genetic alterations identified in ABC-DLBCL [173].

In contrast to ABC-DLBCL, GCB DLBCLs do not acquire highly recurrent mutations in CD79A/B or NF-κB components. Whereas ABC-DLBCL frequently respond to BTK inhibition (see below), GC-DLBCL do not respond and exhibit tonic BCR signaling that does not affect their calcium flux, but acts primarily to activate AKT [174]. Accordingly, forced activation of AKT rescued GCB-DLBCL lines from knockout of the BCR or SYK and CD19, two mediators of tonic BCR signaling [174]. The importance of the oncogenic AKT/PI3K pathway in GCB-DLBCL is evident from the finding that in ~ 55% of patients the tumor suppressor phosphatase and tensin homolog (PTEN), a negative regulator of PI3K, is inactivated. The mechanisms of PTEN inactivation include mutation, deletion or amplification of the miR17–92 microRNA cluster that downregulates PTEN expression [175, 176].

Primary CNS lymphoma (PCNSL), another DLBCL subtype, is an aggressive brain tumor that has a complete response rate of < 40% with methotrexate-based regimens and is subject to late recurrences. Patients showed mutations in the *MYD88, CD79B and CARD11* genes in ~ 58%, ~ 41% and ~ 13% of cases, respectively [177].

### Other B cell malignancies

The hallmark of follicular lymphoma (FL), the (14;18) translocation resulting in BCL2 overexpression, is found in up to ~ 85% of patients. The pathogenesis of FL is complex and involves additional cell-intrinsic genetic changes, frequently including mutations in histone-encoding genes (in ~ 40% of cases), the SWI/SNF complex or the interconnected BCR and CXCR4 chemokine receptor signaling pathways, as well as alterations within the FL microenvironment [178]. The importance of BCR and NF-κB signaling is underscored by the finding of recurrent mutations in the genes encoding C*D22, SLP65/BLNK, PLCγ2, SYK,* PKCβ, *BCL10,* the NF-κB p100 subunit and the deubiquitinating enzyme A20/*TNFAIP3,* which is a negative regulator of NF-κB signaling. In addition, the *HVCN1* gene (coding for a hydrogen voltage-gated proton channel that acts downstream of the BCR and is downregulated in proliferating B cells) is frequently mutated in FL. Interestingly, BTK mutations were found that suggest activation, e.g. the L528 W mutation in the kinase domain, which is associated with resistance to BTK inhibition in CLL (described below), and an in-frame deletion that also alters this amino acid and the adjacent C527. Moreover, two loss-of-function BTK mutations were identified, T117P and R562W, which are also found in XLA patients, but it remains unclear how these mutations contribute to FL pathogenesis [178].

In multiple myeloma (MM), a malignancy of plasma cells in the bone marrow, BTK was shown to be overexpressed, whereby BTK activated AKT signaling, leading to down-regulation of P27 expression and upregulation of key stemness genes [179, 180]. MM cells originate from plasma cells, which do not express surface BCR, and rely for their survival and proliferation on signals from the microenvironment in the bone marrow. BTK may be critical in the MM microenvironment, in particular for secretion of cytokines and chemokines by osteoclasts [181].

Finally, BCR and TLR are thought to be key activation pathways in marginal zone lymphoma (MZL), often associated with chronic inflammation in the context of autoimmunity and/or infection [182], implicating BTK as a potential target. In this context, whole exome sequencing identified recurrent inactivating mutations in Kruppel-like factor 2 (KLF2) which impeded its capacity to suppress NF-κB activation. In addition, recurrent mutations in the TLR/NF-κB pathway were found, affecting e.g. the *MYD88, TRAF3, CARD11, A20/TNFAIP3* and *CARD11* genes [183].

## The BTK inhibitor ibrutinib in clinical studies

Ibrutinib (PCI-32765) is an oral irreversible BTK inhibitor that covalently binds to cysteine at position 481 in the kinase domain and thereby blocks kinase activity [184]. As a result BTK has lost its kinase activity, but Y551 phosphorylation by SYK is not affected. The in vivo effect of ibrutinib was first confirmed in a mouse model of autoimmune disease and in dogs with spontaneous B-cell non-Hodgkin lymphoma, in which it induced objective clinical responses [185].

Efficacy of ibrutinib in a clinical study was first reported in patients with various relapsed/refractory B-cell malignancies, showing clinical safety and promising durable objective responses particularly in CLL and MCL [186]. Responding patients showed sustained reduction in lymphadenopathy, accompanied by transient rise in absolute lymphocyte count, a phenomenon known as lymphocytosis [186]. The next phase Ib/II multicenter trial, with a continuous ibrutinib regimen in relapsed/refractory CLL patients also showed lymphocytosis in the first weeks of treatment, but lymphocyte counts normalized or dropped below baseline after prolonged treatment [11]. Importantly, the overall response rate was ~ 71%, independent of clinical or genomic risk factors.

In a phase II study, patients with relapsed or refractory MCL were treated orally with ibrutinib, resulting in a response rate of ~ 68% [187]. It was subsequently demonstrated that Ibrutinib was also highly active and associated with durable responses in pretreated patients with Waldenström’s macroglobulinemia, whereby MYD88 and CXCR4 mutation status affected the response [188]. Ibrutinib very rapidly received breakthrough designation and was subsequently approved by the Food and Drug Administration (FDA) for the treatment of MCL, CLL and WM between November 2013 and January 2017.

In addition, ibrutinib has also been tested in other B cell malignancies. In line with the possible role of BTK in FL, 6 out of 16 (38%) relapsed/refractory FL patients show response upon ibrutinib treatment [186]. In a phase II study ibrutinib induced durable remissions in ~ 50% of the MZL patients [189]. In a phase I study the majority (77%) of patients with PCNSL show clinical responses to ibrutinib [177]. Table 1 summarizes the data from current clinical trials in various B-cell malignancies.

**Table 1:** Clinical trials with BTK inhibitors in B cell malignancies

Patient population	Therapeutic regimen	Phase	Efficacy	Ref
R/R CLL	Ibrutinib	Ib/II	ORR (71%), PR(20%)	[11]
R/R CLL	Ibrutinib	III	ORR (63%)	[248]
TN CLL	Ibrutinib	Ib/II	ORR (85%), CR(26%)	[199]
TN CLL	Ibrutinib	III	ORR (86%), CR(4%)	[13]
R/R MCL	Ibrutinib	II	ORR (68%), CR(21%)	[187]
R/R MCL	Ibrutinib	III	ORR (72%), CR(19%)	[249]
R/R WM	Ibrutinib	II	ORR(91%), Major response (73%)	[188]
R/R ABC-DLBCL	Ibrutinib	II	ORR (37%)	[196]
R/R CLL	Ibrutinib-Rituximab	II	ORR (95%), CR(8%)	[250]
R/R CLL	Ibrutinib-bendamustine-rituximab	III	ORR (83%), CR(10%)	[251]
R/R MCL	Ibrutinib-Rituximab	II	ORR (88%), CR(44%), PR(44%)	[252]
R/R CLL	Acalabrutinib	I/II	ORR(95%)	[12]
R/R	Acalabrutinib	II	ORR (81%), CR (40%), PR(41%)	[219]
R/R CLL	ONO/GS-4059	I	ORR(96%)	[222]
R/R MCL	ONO/GS-4059	I	ORR(92%)	[222]
R/R non-GCB DLBCL	ONO/GS-4059	I	ORR(92%)	[222]
R/R CLL	BGB-3111	I	ORR(90%)	[221, 253]
R/R MCL	BGB-3111	I	ORR(80%)	[253]
R/R MZL	Ibrutinib	II	ORR(51%)	[254]
R/R FL	Ibrutinib	I	ORR(38%)	[186]

*CLL* Chronic Lymphocytic leukemia, *MCL* Mantle cell lymphoma, *WM* Waldenström’s Macroglobulinemia, *ABC-DLBCL* Activated B-cell Diffuse large B cell Lymphoma, *MZL* Marginal zone lymphoma, *FL* Follicular lymphoma, *R/R* relapsed or refractory, *TN* treatment-naïve, *ORR* overall response rate, *CR* complete response, *PR* partial response, Major response: complete response or at least 50% reduction in serum IgM levels

Several studies were performed to explain the therapeutic mode of action of ibrutinib. In CD40- or BCR-activated CLL cells, ibrutinib reduced survival by abrogating downstream pathways including ERK, PI3K and NF-кB [141]. Ibrutinib inhibited migration of CLL cells towards chemokines such as CXCL12 and CXCL13, suggesting that treatment inhibits homing and retention of malignant cells in their survival niches [77]. Ibrutinib was also found to reduce secretion of BCR-dependent chemokines CCL3 and CCL4 [142]. Another key effect was that it inhibited integrin α4β1-mediated adhesion of CLL cells to fibronectin and VCAM1 [146] and thus interaction with the tumor microenvironment [146]. Therefore, ibrutinib apparently works by a dual mechanism, by inhibiting intrinsic B cell signaling pathways to compromise their proliferation and survival as well as by disrupting tumor-microenvironment interactions. Importantly, both in CLL and MCL ibrutinib treatment induces a redistribution lymphocytosis, a transient rise of leukemic cells in the circulation and a concomitant rapid reduction of these cells at the affected tissue sites. In contrast to classical cytotoxic chemotherapy, ibrutinib does not cause tumor lysis syndrome, which is a common complication of cancer therapy because of metabolic disturbances when large numbers of tumor cells die quickly. Therefore, most likely the displacement of B cells from nurturing tissue niches because of inhibition of integrin-mediated retention of leukemic cells, is an important mechanism of action of ibrutinib, rather than robust inhibition of survival of malignant B cells [190]. As a result, leukemic cells undergo ‘death by neglect’, because their mobilization induces ‘homelessness’ (anoikis), a form of programmed cell death [191, 192].

Despite impressive clinical success of ibrutinib, its curative potential in B cell malignancies is not established yet, as ibrutinib is often prescribed as life-long therapy. Importantly, continuous therapy may lead to selection or outgrowth of resistant clones, as described in a subset of patients who relapse upon ibrutinib therapy. Two important therapy-associated resistance mechanisms have been identified, involving BTK C481S mutation (the site of action of Ibrutinib) or activating mutations in PLCy2 (R665W, S707Y and L845F) [193, 194]. Recently another BTK mutation, T316A in the SH2 domain, was described, as well as clonal evolution underlying leukemia progression in patients with ibrutinib-relapsed CLL [195]. In addition, missense mutation within the coiled-coil domain of CARD11 (R179Q) have been shown to promote BTK-independent activation of NF-κB and thus ibrutinib resistance in DLBCL, MCL and PCNSL [177, 196, 197]. Furthermore, an activating mutation in BTK (L528 W) that confers resistance to ibrutinib treatment has been found in CLL and FL [178, 198].

In clinical trials the adverse events were mostly limited to grade 1 or 2 in severity, but in some cases side-effects led to discontinuation of the therapy [199–201]. Because ibrutinib treatment has a considerable high risk of bleeding in treated patients, concomitant anti-coagulation (~ 11%) and antiplatelet (~ 34) use is common and ~ 3% of the patients were reported to have major bleeding events [202]. Atrial fibrillation has been reported in up to 16% of patients taking ibrutinib, whereby stroke prevention poses a challenge because of the increased bleeding risk. Therefore, close monitoring is recommended, especially during the first 6 months of ibrutinib therapy [203]. Although the occurrence of atrial fibrillation might possibly be related to inhibition of the BTK-regulated PI3K/AKT pathway in cardiac myocytes [204], the mechanisms involved remain largely unidentified.

Three year follow-up of ibrutinib-treated CLL patients showed that prolonged treatment was associated with improvement in response quality (the ORR increased to > 90%) and durable remission, while toxicity including cytopenia, fatigue, and infection diminished. Moreover, progression remains uncommon [205]. Findings from the longest follow-up reported to date, evaluating up to 5 years of ibrutinib in CLL patients, show that it is relatively safe and effective, with ~ 89% of treatment-naïve and relapsed patients experiencing a response to the therapy [206].

Part of the toxicities and side effects of ibrutinib can be explained by its non-specific nature: ibrutinib is not an exclusive inhibitor of BTK and off-target inhibition includes kinases that contain a cysteine residue aligning with Cys-481 in BTK. These include other TEC-family kinases (ITK, BMX, TEC), as well as epidermal growth factor receptor (EGFR), T-cell X chromosome kinase (TXK) and Janus Kinase 3 (JAK3) [12, 185, 207]. In this context, it is of note that the bleeding risk in patients receiving ibrutinib was thought to relate to off-target inhibition of TEC [12]. BTK is expressed in platelets where it is important for signaling via the collagen receptor glycoprotein VI (GPVI); platelets from XLA patients display diminished aggregation, dense granule secretion and calcium mobilization in response to collagen and C-reactive protein [208]. Nevertheless, XLA patients do not have an increased risk of bleeding [209]. Findings by *Bye* et al. indicated that both BTK and TEC – although required for GPVI-mediated platelet aggregation – are redundant for platelet adhesion to collagen and thrombus formation [210]. Rather, ibrutinib but not the more selective BTK inhibitor acalabrutinib (see below) inhibits SRC family kinases that have a critical role in platelet function [210]. These findings explain why in contrast to ibrutinib, treatment with acalabrutinib was not associated with major bleeding events [12].

A recent systematic review of infectious events with ibrutinib in the treatment of B cell malignancies provided evidence for infection-related complications in ~ 50% of patients taking ibrutinib, whereby ~ 20% of patients developed pneumonia due to opportunistic pathogens [211]. Hereby, data suggest that these events may involve inhibition of both BTK and its closely related family member ITK. On the other hand, it was shown that ibrutinib treatment increased the in vivo persistence of both CD4^+^ and CD8^+^ activated T cells and diminished the immune-suppressive properties of CLL cells. As these effects were not seen with more specific BTK inhibitor acalabrutinib that lacks ITK inhibitory activity (see below), it was concluded that the T cell expansion is unlikely to be caused by BTK inhibition [212]. Rather, ibrutinib treatment of activated T cells diminishes activation-induced cell death by targeting ITK, a finding also reported in murine models of ITK deficiency. However, both inhibitors reduced the expression of the inhibitory co-receptors programmed cell death protein 1 (PD-1) and cytotoxic T-lymphocyte-associated protein 4 (CTLA4) on T cells, as well as expression of the immunosuppressive molecules CD200, B- and T-lymphocyte attenuator (BTLA) and IL-10 by CLL cells [212]. Therefore, ibrutinib likely diminishes the immune-suppressive properties of CLL cells through both BTK-dependent and ITK-dependent mechanisms.

Inhibition of BTK and ITK with ibrutinib was shown to be effective in the prevention of chronic graft-versus-host (GvH) disease following allogeneic hematopoietic stem cell transplantation (SCT) in several mouse models [213, 214]. Accordingly, also studies in patients with relapsed CLL following SCT support that ibrutinib augments the GvH versus-leukemia (GVL) benefit likely through ITK inhibition [215]. In particular, it was shown that ibrutinib selectively targeted pre–germinal B cells and depleted Th2 helper cells, whereby these effects persisted after drug discontinuation.

Taken together, these findings provide a rationale for combination immunotherapy approaches with ibrutinib in CLL and other cancers.

## Ibrutinib in combination therapies and second generation BTK inhibitors

The finding of ibrutinib resistance, together with multiple modes of action and the microenvironmental dependence of B-cell malignancies, has fueled the development of novel combination strategies. With the aim to achieve deeper remissions within a short treatment time, many ibrutinib combination therapies are currently considered (Table 2). Hereby, ibrutinib treatment forces egress of malignant B cells out of their protective niches into circulation, where they become vulnerable to direct cytotoxic activity of either chemotherapy, an inhibitor of the pro-survival protein Bcl-2, or antibody mediated cytotoxicity (ADCC) of anti-CD20 antibody therapy.

**Table 2:** Overview of Ibrutinib in combination therapies

Combination	Disease	Model	Rationale	Effect	Reference
γ-secretase inhibitors (crucial protease in Notch signaling)	CLL	CLL patient cells	NOTCH1 signaling is related to resistance to therapy in B-CLL.	Combination therapy showed enhanced cytotoxicity and reduced CXCR4 expression and functions (response to SDF-1α)	[255]
Histone Deacetylase (HDACs) Inhibitor	CLL	- MCL cell line - mice engrafted with TCL-1 splenocytes	HDACs increase transcription of miRNA that repress BTK	HDAC induced increase in target miRNA and a decrease in BTK RNA; combination exhibited higher cytotoxicity than either drug alone; reduction of p-BTK and total BTK protein.	[69]
Anti-CD19 CAR T Cells (CART19)	MCL	MCL Xenograph model	Efficient B cell depletion	Long-term remission in 80–100% of mice (treated with CART19 only: 0–20% of mice)	[245]
Ethacridine (Poly(ADP-ribose) glycohydrolase inhibitor)	AML	SCID mice injected s.c. with OCI-AML2 cells	Result of a drug screening	High decrease of OCI-AML2 cell growth (more than with either drug alone). Suggested mechanism: increased intracellular ROS production in cells treated with combination.	[256]
ND-2158 (IRAK4 inhibitor)	ABC-DLBCL	- ABC-DLBCL cell lines OCI-Ly10 and TMD8 - OCI-Ly10 xenografts	MYD88-IRAK4 signaling is important for ABC- DLBCL viability	Combination was more effective than ND-2158 alone in inhibiting IKK activity, enhancing apoptosis, and blocking tumor growth in mice.	[257]
PU-H71 (Binds to tumor enhanced HSP90 complexes)	ABC-DLBCL	DLBCL cell lines (HBL-1 and TMD8)	teHSP90 complexes are associated with tumor survival.	PU-H71 disrupts teHSPP90 (but not house-keeping fractions associated with HSP90). Synergistic effect, with ~ 95% tumor growth inhibition; decreased NF-kB activity	[258]
TP-0903 (AXL inhibitor)	CLL	Patient CLL cells prior to or after ibrutinib therapy	AXL contributes to oncogenic survival in CLL.	TP-093 disrupts the activity of AXL; Induction of cell-death in a dose-dependent fashion	[259]
B-PAC-1 (pro-caspase activating compound)	CLL	B cells from patients on ibrutinib therapy	B-PAC activates caspases dimers	Induced cytotoxicity in leukemic cells	[259]
Carfilzomib (proteasome inhibitor)	CLL	Primary CLL patient samples MEC-1 and MEC2 cell lines	Upregulation of pro-apoptotic transcription factor CHOP	Combination showed an additive cytotoxic effect; Carfilzomib induced a pro-apoptotic response involving Noxa, MCL-1, Bax, and Bak and intrinsic and extrinsic caspase pathways	[260]
Selinexor (Exportin inhibitor)	CLL	Primary CLL patient samples	Selinexor disrupts BCR signaling via BTK depletion	Combination showed synergistic cytoxicity. Selinexor overcomes resistance to Ibrutinib (also in patient cells with C481S mutation)	[261]
Anti-PDL1 antibody (Negative regulator of T cell function)	B cell lymphoma (A20)	- BALB/c mice inoculated with A20 B cells - A20 B cells are resistant to Ibrutinib	Blocking immune checkpoints can enhance the anti-tumor response	Anti-PDL-1 treatment alone delayed tumor growth and slightly increased mouse survival Combination with anti-PDL-1 cured ~ 50% of the mice, delayed tumor growth and prolonged survival in the remaining mice, and increased IFN-γ producing T-cells	[243]
ABT-199 (BCL-2 antagonist)	CLL	Ex vivo samples from CLL patients on ibrutinib	CLL samples show enhanced BCL-2 expression	Ibrutinib enhances ABT-199 cytotoxicity, both in unstimulated and in αIgM-stimulated CLL cells from. ABT-199 action correlated with a decline in expression of anti-apoptotic MCL-1	[262]
ABT-199 (BCL-2 antagonist)	MCL	CCMCL1 MCL cell line	MCL cells show enhanced BCL-2 expression	Combination results in decrease of p-BTK and p-AKT. Downregulation of both BCL2 and MCL1. ABT-199 and Ibrutinib target non-overlapping pathway s	[263]
Bortezomib (proteosome inhibitor) and lenalidomide chemotherapy	MM	Cells from MM patients and MM cell lines	Blocking BTK to downregulate NF-kB activation and cell survival	Ibrutinib increased the cytotoxicity of bortezomib and lenalidomide in both patient cells and cell lines	[264]
CpG (TLR9 ligand)	B-cell lymphoma	Murine pre-B cell (H11) and B cell lymphoma lines (BL3750, A20)	CpG activates APCs and thereby induces T cell activation	Combination of ibrutinib and intratumoral CpG resulted in tumor regression and resistance, whereby IFNy-producing CD4 and CD8 T are essential	[265]
Sudemycin D1 (spliceosome modulator)	CLL	Primary CLL cells (from SF3B1-unmutated and mutated cases)	*SF3B1* is frequently mutated in CLL, and correlates with aggressiveness	Combination results in enhanced apoptosis of M-CLL and U-CLL. Effect is related to IBTK splicing. Sudemycin D1 downregulates anti-apoptotic MCL-1 through alternative splicing	[266]
BAY80–6946 (PIK3 inhibitor) INK128 (mTOR inhibitor)	PCNSL	- Xenograft model from CD79B-mutant biopsies	CARD11 domain mutations increase the activity of the PIK3-mTOR axis	In cell lines, cell death was induced with both combinations of drugs	[177]
Idelalisib (PI3K inhibitor)	DLBCL	- Cell lines. - Mouse TMD8 xenograft model	PI3K is upstream regulator of NF-кB pathway.	Cell lines: combination induced 50% apoptosis and inhibited signaling (more than either drug individually). Mouse xenograft: Significant tumor regression	[267]^1^
Idelalisib (PI3K inhibitor)	MCL	MCL cell lines	A more robust blockage of BCR signaling	Inhibition of BCR-stimulated integrin- mediated adhesion; stronger inhibition of adhesion compared to each drug alone	[146]^1^

^1^In this study, also ONO/GS-4059, the phosphoinositide-dependent kinase-1 inhibitor GSK2334470 and the AKT inhibitor MK-2206 were investigated

*CLL* Chronic Lymphocytic leukemia, *MCL* Mantle cell lymphoma, *AML* Acute Myeloid Leukemia, *ABC-DLBCL* Activated B-cell Diffuse large B cell Lymphoma, *MM* Multiple Myeloma, *PCNSL* Primary central nervous system lymphoma

Side-effects associated with off-target kinase inhibition may limit the use of ibrutinib as therapeutic agent (as discussed above). Ibrutinib can antagonize rituximab-induced ADCC due to inhibition of its family member ITK in NK cells, further limiting its use in combination regimens [216]. Therefore, many efforts have focused on developing highly selective BTK inhibitors, of which three have reached advanced stages of clinical development [217].

### Acalabrutinib (ACP-196)

This highly selective irreversible BTK inhibitor has significantly less off-target kinase activity [207]. Acalabrutinib also binds C481 and lacks irreversible targeting to alternative kinases, such as EGFR, ITK, TXK, SRC family kinases and JAK3. The first pre-clinical study in canine models of Non-Hodgkin B-cell lymphoma demonstrated enhanced in vivo potency compared to ibrutinib [218]. In a phase I/II clinical trial in patients with relapsed/refractory CLL the overall response rate was ~ 95% and in patients with del(17)(p13.1) this was 100%, with a median follow-up up ~ 14 months [12]. No dose-limiting toxicities, episodes of atrial fibrillation, or bleeding-related events have been reported to date. To investigate the superiority of either inhibitor, a phase III trial for direct comparison of ibrutinib with acalabrutinib in R/R CLL patients is currently ongoing (NCT02477696). Additionally, in a phase II trial in patients with relapsed/refractory MCL, acalabrutinib induced an overall response of ~ 81% with ~ 40% patients achieving a complete response [219]. This led to accelerated FDA approval of acalabrutinib in MCL [220].

### BGB-3111

Another selective inhibitor of BTK kinase activity with superior oral bioavailability and higher selectivity than ibrutinib is BGB-3111, which was shown to inhibit proliferation of several MCL and DLBCL cell lines. Due to weaker ITK inhibition, BGB-3111 was at least 10-fold weaker than ibrutinib in inhibiting rituximab induced ADCC. When 45 CLL patients were treated on a phase I/II study, therapy was well tolerated and was associated with a response rate of ~ 90% after a follow-up of 7.5 months and no cases of disease progression or Richter’s transformation [221] (see also Table 1).

### Ono/GS-4059

In vivo efficacy of this compound was initially described in an ABC-DLBCL xenograft model and in vitro anti-proliferative effects in DLBCL, FL, MCL and CLL cell lines were described [222]. Early-phase clinical trial data in patients with several B-cell malignancies include clinical responses in patients with high-risk CLL genetics (Table 1).

## Role of BTK in the tumor microenvironment

Inhibition of BTK has now also extended into the field of solid tumors, following insights into the role of BTK in various cells of the tumor microenvironment and in non-hematological tumor cells when ectopically expressed. An understanding of the diverse roles of BTK in non-lymphocytic cells will be pivotal in the development of novel treatment combinations for haematopoietic and solid tumors.

BTK is involved in TLR- and Fc-receptor mediated activation, maturation, migration and survival of myeloid cells [223, 224]. However, the role of BTK identified is dependent on cell type investigated, the nature of activating stimuli, the model used (in vivo or in vitro) and the species investigated, i.e. mouse or human. Analyses in various mouse models and in vitro studies with myeloid cells from XLA-patients clearly implicate BTK in TLR4/8/9-signaling, and possibly others [79, 225–227]. However, data are often conflicting, e.g. TLR8-induced IL-6 production by BTK-deficient DCs were reported to be impaired [226], enhanced [228], or unaffected [229]. Also TLR4/7/8-induced TNFα was reported to be reduced [226, 229] or enhanced [228].

Of further relevance in the context of the tumor microenvironment is the polarization status of macrophages, with M1 macrophages displaying a pro-inflammatory anti-tumor phenotype and M2 macrophages being immunosuppressive [22]. Whereas one study indicated an M2-skewing of BTK-deficient macrophages [230], recently in a pancreatic cancer mouse model an M1-skewing of intratumoral macrophages was found following ibrutinib treatment [231]. In contrast, ibrutinib induced M1 to M2-skewing of nurse-like cells, which show properties of tumor-associated macrophages, accompanied by impaired phagocytosis, increased IL-10 production mediating pro-survival signals in CLL [232]. It remains unknown what causes these incongruences in BTK-dependent myeloid polarization, however it is conceivable that the different roles of BTK in a complex ecology of tumor-infiltrating cells and the limited specificity of ibrutinib contribute to the conflicting findings.

In solid tumors, chronic deposition of immune complexes foster carcinogenesis due to chronic inflammation, angiogenesis and M2 macrophage polarization in response to activating Fc-receptor ligation on myeloid cells [231, 233, 234]. Interestingly, inhibiting BTK during Fc-receptor stimulation of macrophages in vitro using Ibrutinib prevented M2-skewing [231].

Granulocytes and their immature immune-suppressive counterparts, myeloid derived suppressor cells (MDSC), are strongly implicated in tumor progression, rendering them important candidates for therapy [235]. Although loss of BTK in XLA neutrophils does not impair functional TLR responses [236], the numbers of circulating granulocytes are reduced in XLA-patients and BTK-deficient mice [237–239]. Moreover, BTK-deficient neutrophils manifest increased sensitivity to apoptosis, decreased maturation, differentiation, trafficking and impaired functionality including reactive oxygen species (ROS) production [238–241]. Likewise, ibrutinib treatment inhibited the generation, migration, TNFα and ROS-production of MDSCs both in vitro and in solid tumor mouse models [242]. Ibrutinib treatment partially alleviated MDSC-mediated CD8^+^ T-cell suppression and enhanced anti-PD-L1 therapy efficacy in a breast cancer model. BTK inhibition in granulocytes and MDSCs in solid tumors may therefore be important in the development of effective combination therapies.

## BTK inhibition in solid malignancies

Ectopic BTK expression has been observed in various solid tumors, whereby evidence is accumulating for its involvement in oncogenesis [24–27]. These pre-clinical findings have led to the initiation of several early phase I/II clinical trials in which BTK inhibition monotherapy is evaluated in advanced ovarian, colorectal, prostate and brain cancer patients(Table 3).

**Table 3:** Clinical trials with BTK-inhibitors in solid tumors

BTK-inhibitor	Tumor Type	Treatment combination	Phase Trial	Status	NCT#
Ibrutinib	Pancreatic Cancer	nab-paclitaxel and gemcitabine	ll/lll	Ongoing, Not Recruiting	NCT024B6668
Ibrutinib	Pancreatic Cancer	nab-paclitaxel and gemcitabine	I	Recruiting	NCT02562898
Ibrutinib	Renal/Urethelial/Gastric/Colorectal Cancer	chemotherapy/small molecule	Ib/II	Recruiting	NCT02599B24
Ibrutinib	HER2/MYC + Oesophageal Cancer		I	Recruiting	NCT02884453
Ibrutinib	EGFR + Non-small Cell Lung Cancer (NSCLC)		I/ll	Ongoing, Not Recruiting	NCT02321540
Ibrutinib	NSCLC/Breast Cancer/Pancreatic Cancer	Durvalumab (anti-PD-L1)	I/ll	Completed	NCT02403271
Ibrutinib	Pancreatic Neuro-endocrine tumors (pNET) /metastatic Carcinoid		II	Recruiting	NCT02575300
Ibrutinib	Stage IV Cutaneous Melanoma		II	Recruiting	NCT02581930
Ibrutinib	Metastatic Renal Cancer	Nivolumab (anti-PD-L1)	lb./ll	Recruiting	NCT02899078
Ibrutinib	Localized Prostate Cancer		I/II	Recruiting	NCT02643667
Acalabrutinib	Advanced/Metastatic Pancreatic Cancer	Pembrolizumab (anti-PD-L1)	II	Ongoing, Not Recruiting	NCT02362048
Acalabrutinib	Recurrent Ovarian Cancer	Pembrolizumab (anti-PD-L1)	II	Ongoing, Not Recruiting	NCT02537444
Acalabrutinib	Glioblastoma		lb/ll	Recruiting	NCT02586857
Acalabrutinib	NSCLC	Pembrolizumab (anti-PD-L1)	II	Ongoing, Not Recruiting	NCT02448303
Acalabrutinib	Head and Neck Squamous Cell Carcinoma (HNSCC)	Pembrolizumab (anti-PD-L1)	II	Ongoing, Not Recruiting	NCT02454179
Acalabrutinib	Platinum-resistant Urethelial (Bladder) Cancer	Pembrolizumab (anti-PD-L1)	II	Ongoing, Not Recruiting	NCT02351739,

Also in BTK-negative solid tumors that do not express BTK, its inhibition may hold promise as multiple cell types in the tumor microenvironment are regulated by BTK. Inhibition of BTK in pre-clinical models of pancreatic cancer, breast cancer and BTK-negative colon cancer have shown only marginal improvement of survival as monotherapy, but when combined with chemo- or immunotherapy, survival was greatly enhanced [231, 242, 243]. This has sparked the emergence of several trials investigating the safety and efficacy of ibrutinib or acalabrutinib, in combination with conventional PD-1/PD-L1 checkpoint inhibition therapy (Table 3).

Given that ibrutinib shows off-target inhibition of JAK3, ITK and EGFR [185, 207], it can be used to target oncogenic pathways other than BTK in tumor cells and as a T-cell modulator in combination immunotherapy [243–246]. Hereby, ibrutinib treatment increased cellular persistence and decreased expression of co-inhibitory surface molecules on Chimeric antigen receptor (CAR) T cells in models of CLL and MCL [245, 246]. Whether in these studies ibrutinib acts on ITK in (CAR) T cells, on BTK in the malignant cells or other kinases remains undetermined. Paradoxically, inhibiting ITK in T cells may be efficacious in cancer, as this may enhance Th1-skewing of CD4^+^ T-cells and thereby improved memory formation and functionality of CD8^+^ T-cells, potentially leading to improved anti-tumor immunity [243, 247]. These potentially beneficial off-target effects of ibrutinib may be lost in the highly specific BTK-inhibitors that are currently being evaluated.

## Conclusions

Targeting of BTK, which has a central role in several signaling pathways in B cells, particularly the BCR, has shown impressive efficacy as therapeutic option for various B cell malignancies in clinical trials. Much progress has been made in recent years in defining the complex mechanisms of action of BTK inhibition. These involve intrinsic signaling pathways in leukemic cells that are central to cellular survival, proliferation and - most importantly - retention in supportive microenvironments. Moreover, BTK inhibition shows promise as a therapy that influences crucial immune cells in the tumor microenvironment. Because data from BTK-deficient or inhibitor-treated myeloid cells in the context of cancer are scarce, it is not clear whether BTK inhibition by e.g. ibrutinib is based on its specificity for BTK in particular myeloid cells and/or due to off-target effects in signaling pathways in CD4^+^ or CD8^+^ T cells. Of note, because in CLL ibrutinib treatment diminished the immunosuppressive properties of malignant cells through BTK-dependent and BTK-independent mechanisms (probably via ITK inhibition) [212], it will be interesting to observe whether the same level of anti-tumor efficacy is maintained by specific BTK inhibition alone. It is very well conceivable that for particular malignancies it may be advantageous to use BTK inhibitors that show additional specificity for related kinases.

Although the efficacy of BTK inhibition as a single agent therapy is strong, it has been shown that resistance may develop and now a broad range of studies focus on development of effective combination therapies to improve clinical responses. The identification of differences in efficacy and toxicity profiles between available BTK inhibitors awaits direct comparative studies. In this context, design of treatment strategies will depend on detailed analyses of clinical responses, resistance development, toxicity and quality of life for individual BTK inhibitors in combination therapies in relation to the various malignancies and patient subgroups.

## Abbreviations

ABC-DLBCL: Activate B-cell diffuse large B-cell lymphoma
BCR: B cell receptor
BTK: Bruton’s tyrosine kinase
CLL: Chronic lymphocytic leukemia
CR: Complete response
DLBCL: Diffuse large B cell lymphoma
ERK: Extracellular signal-regulated kinase
GC: Germinal center
ITAM: Immunoreceptor tyrosine-based activation motif
ITIM: Immunoreceptor tyrosine-based inhibitory motif
MCL: Mantle cell lymphoma
M-CLL: Mutated chronic lymphocytic leukemia
MYD88: Myeloid differentiation primary response 88
NFAT: Nuclear factor of activated T cells
NF-κB: Nuclear factor kappa-light-chain-enhancer of activated B cells
ORR: Overall response rate
OS: Overall survival
PCNSL: Primary central nervous system lymphoma
PD-1: Programmed cell death protein 1
PI3K: Phosphatidyl-inositol-3-kinase
PIP3: Phosphatidylinositol-3,4,5-triphosphate
PLCγ: Phospholipase C γ
PR: Partial response
SHIP1: SH2-domain containing inositol polyphosphate 5’phosphatase-1
SHM: Somatic hypermutation
SHP1: SH2 domain containing protein tyrosine phosphatase-1
SYK: Spleen tyrosine kinase
TLR: Toll-like receptor
U-CLL: Unmutated chronic lymphocytic leukemia
WM: Waldenström’s Macroglobulinemia
