# Correction to: Role of Bruton’s tyrosine kinase in B cells and malignancies

**DOI:** 10.1186/s12943-019-1009-z

**Published:** 2019-04-03

**Authors:** Simar Pal Singh, Floris Dammeijer, Rudi W. Hendriks

**Affiliations:** 1 Department of Pulmonary Medicine, Room Ee2251a, Erasmus MC Rotterdam, PO Box 2040, NL 3000 CA Rotterdam, The Netherlands; 2 Department of Immunology, Rotterdam, The Netherlands; 3 Post graduate school Molecular Medicine, Rotterdam, The Netherlands; 4 Erasmus MC Cancer Institute, Erasmus MC, Rotterdam, The Netherlands

**Correction to: Mol Cancer**

**https://doi.org/10.1186/s12943-018-0779-z**

Following publication of the original article [1], the authors reported an error in Table 1. Incorrect value was placed under Efficacy (column), R/R non-GCB DLBCL (row).The value 92% was captured instead of 35%. Corrected table is shown below. The authors would like to apologize for this error.

**Table 1:** Clinical trials with BTK inhibitors in B cell malignancies

Patient population	Therapeutic regimen	Phase	Efficacy	Ref
R/R CLL	Ibrutinib	Ib/II	ORR (71%), PR (20%)	[11]
R/R CLL	Ibrutinib	III	ORR (63%)	[248]
TN CLL	Ibrutinib	Ib/II	ORR (85%), CR (26%)	[199]
TN CLL	Ibrutinib	III	ORR (86%), CR (4%)	[13]
R/R MCL	Ibrutinib	II	ORR (68%), CR (21%)	[187]
R/R MCL	Ibrutinib	III	ORR (72%), CR (19%)	[249]
R/R WM	Ibrutinib	II	ORR (91%), Major response (73%)	[188]
R/R ABC-DLBCL	Ibrutinib	II	ORR (37%)	[196]
R/R CLL	Ibrutinib-Rituximab	II	ORR (95%), CR (8%)	[250]
R/R CLL	Ibrutinib-bendamustine-rituximab	III	ORR (83%), CR (10%)	[251]
R/R MCL	Ibrutinib-Rituximab	II	ORR (88%), CR (44%), PR (44%)	[252]
R/R CLL	Acalabrutinib	I/II	ORR (95%)	[12]
R/R	Acalabrutinib	II	ORR (81%), CR (40%), PR (41%)	[219]
R/R CLL	ONO/GS-4059	I	ORR (96%)	[222]
R/R MCL	ONO/GS-4059	I	ORR (92%)	[222]
R/R non-GCB DLBCL	ONO/GS-4059	I	ORR (35%)	[222]
R/R CLL	BGB-3111	I	ORR (90%)	[221, 253]
R/R MCL	BGB-3111	I	ORR (80%)	[253]
R/R MZL	Ibrutinib	II	ORR (51%)	[254]
R/R FL	Ibrutinib	I	ORR (38%)	[186]

*CLL* Chronic Lymphocytic leukemia, *MCL* Mantle cell lymphoma, *WM* Waldenström’s Macroglobulinemia, *ABC-DLBCL* Activated B-cell Diffuse large B cell Lymphoma, *MZL* Marginal zone lymphoma, *FL* Follicular lymphoma, *R/R* relapsed or refractory, *TN* treatment-naïve, *ORR* overall response rate, *CR* complete response, *PR* partial response, Major response: complete response or at least 50% reduction in serum IgM levels
